# Gender medicine and sleep disorders: from basic science to clinical research

**DOI:** 10.3389/fneur.2024.1392489

**Published:** 2024-07-10

**Authors:** Elisa Perger, Rosalia Silvestri, Enrica Bonanni, Maria Caterina Di Perri, Mariana Fernandes, Federica Provini, Giovanna Zoccoli, Carolina Lombardi

**Affiliations:** ^1^Istituto Auxologico Italiano, IRCCS, Sleep Disorders Center and Department of Cardiovascular, Neural and Metabolic Sciences, San Luca Hospital, Milan, Italy; ^2^Sleep Medicine Center, Neurophysiopathology and Movement Disorders Unit, Department of Clinical and Experimental Medicine, University of Messina, AOU “G. Martino”, Messina, Italy; ^3^Sleep Disorder Center, Neurology Unit, Azienda Ospedaliero-Universitaria Pisana and Department of Clinical and Experimental Medicine, University of Pisa, Pisa, Italy; ^4^Epilepsy Centre, Department of Systems Medicine, University of Rome “Tor Vergata”, Rome, Italy; ^5^Neurology Unit, University Hospital of Rome “Tor Vergata”, Rome, Italy; ^6^IRCCS, Istituto delle Scienze Neurologiche di Bologna, Bologna, Italy; ^7^Department of Biomedical and Neuromotor Sciences, Università di Bologna, Bologna, Italy; ^8^Department of Medicine and Surgery, University of Milano-Bicocca, Milan, Italy

**Keywords:** sleep medicine, women, restless legs syndrome (RLS), sleep apnea, narcolepsy, animal models, gender, sleep disordered breathing

## Abstract

Several pivotal differences in sleep and sleep disorders are recognized between women and men. This is not only due to changes in hormonal balance during women's reproductive life, such as in pregnancy and menopause. Women are more likely to report insomnia and non-specific symptoms of apneas, such as fatigue or mood disturbance, compared to men. Thus, it is important for clinicians and researchers to take sex and gender differences into account when addressing sleep disorders in order to acknowledge the biology unique to women. We present a narrative review that delves into the primary sleep disorders, starting from basic science, to explore the impact of gender differences on sleep and the current status of research on women's sleep health.

## Introduction

The study of gender differences in sleep disorders, and more generally in neuroscience, has only recently garnered attention. While there is extensive knowledge on the physical variances between men and women, there has been a dearth of research on disparities in brain function and behavior. Historically, neuroscientists believed that the cognitive and behavioral differences between genders were primarily a result of cultural and social factors, with biological differences being linked to different hormonal disparities. In the 1960's, Seymour Levine published an article summarizing the existing knowledge on sex-related brain differences, highlighting variations in mating behaviors between male and female rats and examining the impact sex hormones on these behaviors (1). Interestingly, the only brain area considered was the hypothalamus, for its role as a regulator of hormone production. Hence, sex differences in the brain referred exclusively to sexual behaviors, sex hormones and the hypothalamus. This attitude also involved basic scientists, who until recently seldom used female rodents, believing that hormonal variations introduced an unnecessary confounding bias in their experiments. Hormones, in fact, were expected to act on brains that were presumed to be identical in males and females.

In modern times, it is understood that there are significant distinctions in the structure and function of male and female brains, leading to the recognition of the brain as a sexually dimorphic organ. The relationship between sleep disorders and gender is not just a matter of who snores more; there are also dramatic differences in the way men and women experience sleep and its disorders. Women typically experience more slow wave sleep, but also endure more sleep disturbances compared to men. Research also suggests that women frequently exhibit more severe symptoms of depression, difficulty sleeping, and insomnia. Furthermore, women are often required to sleep in a separate room because of their male partners' sleep disorders (2, 3).

Despite previous attempts to address disparities in treating sleep disorders in women and men, there are still substantial gaps in knowledge and a lack of awareness among the research community. Scientists and physicians must prioritize sex and gender differences in their research on sleep disorders to duly acknowledge the distinct biology of women. Differences in sleep behavior and sleep disorders may not only be driven by biological factors, but also by gender-specific differences in how symptoms are communicated by women and men. Women may experience changes in sleep due to varying hormonal levels during different stages of their reproductive life, like pregnancy and menopause (4). In clinical populations, women are more likely to present with insomnia than men, although their sleep quality may be better preserved. Women with sleep apnea may not exhibit the typical symptoms of snoring and sleepiness that men often report; instead, they are more likely to report symptoms such as asthenia, tiredness, or mood disorders. Thus, gender bias has resulted in delayed diagnosis of important sleep disorders such as narcolepsy, obstructive sleep apnea syndrome (OSAS) or restless legs syndrome (RLS) in women. As a first step toward individualized medicine, identifying the underlying gender-related factors in sleep research has the potential to accelerate improved care for both men and women.

**Figure 1:**
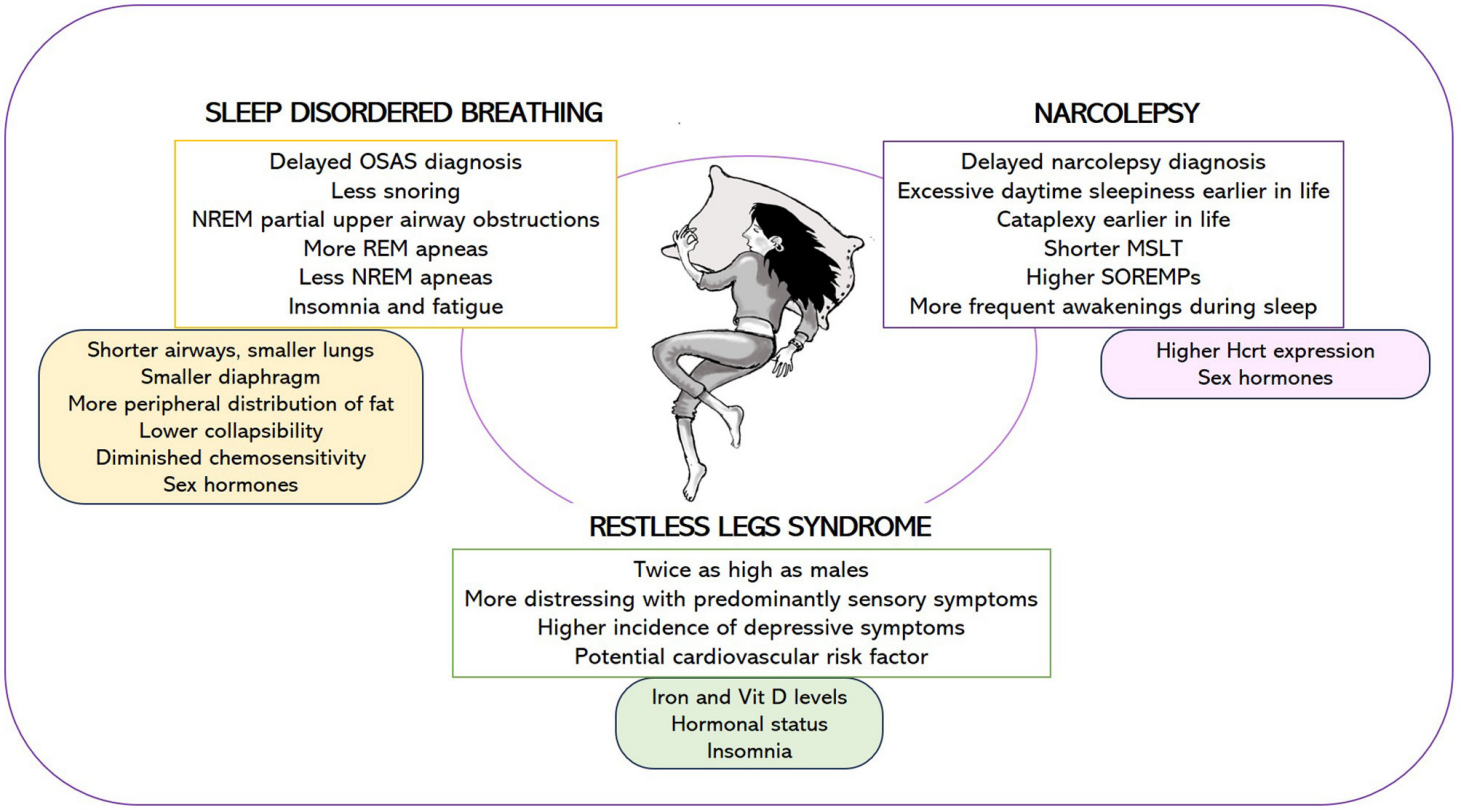
Female-specific features of narcolepsy, sleep disordered breathing, and restless legs syndrome **(upper box)** and potential causes underlying these gender differences **(lower box)**. OSAS, obstructive sleep apnea syndrome; MSLT, mean sleep latency during the Multiple Sleep Latency Test; SOREMPs, number of sleep onset REM periods; Hcrt, hypocretin.

To comprehend how gender impacts sleep and the current status of research on women's sleep health (summarized in Figure 1), we present a narrative review that explores common sleep disorders while drawing insights from murine models. A search of relevant medical literature in the English language was conducted using the Medline/PubMed and EMBASE databases. Our aim is to lead the reader through the extensive field of sleep medicine, with a specific focus on gender differences. We believe that focused reviews of basic and clinical research on challenges posed by gender-related sleep problems will enable the identification of knowledge gaps and the development of research-related recommendations.

## Gender differences in sleep disorders: experience from murine models

The growing awareness of the biological differences present in the brains of males and females has fueled a significant surge in studies examining sleep disorders in animal models. Rodent models are particularly valuable in researching gender disparities, and in neuroscience in general, as mice and rats have a brief life span. This enables us to monitor the development of a disease over their lifetime or investigate its association with the aging process. Mice can also be genetically modified to serve as models for specific human diseases. Moreover, animals enable the use of invasive methods that are not suitable for human subjects. Thus, animal studies have been instrumental in shaping our understanding of the biological factors contributing to gender disparities. Lastly, employing animal models can eliminate numerous interfering variables, including genetic background, environmental factors, diet, medication usage, and lifestyle.

In this context, animal models are increasingly being utilized to study gender differences in various sleep disorders. Among these, sleep-disordered breathing (SDB) has received special attention. Gender studies in mice models seemed suitable because the occurrence of sleep apnea, and sleep-disordered breathing in general, varies between men and women, with higher rates in men (5). However, the difference might be attributable to the fact that these disorders are often unrecognized or underdiagnosed in women, who present partly atypical symptoms (6). According to the Society of Women's Health Research (SWHR), only 1 in 4 women with OSAS are diagnosed (7).

Knowledge of the biological mechanisms underlying gender differences in sleep-disordered breathing is still very limited, and the use of the mouse model represents a valid aid for the study of these mechanisms, as well as for the development of innovative therapies.

A recent study revealed that male mice with diet-induced obesity experience SDB, while obese female mice show a decrease in the severity of sleep apnea, along with reduced CO_2_ sensitivity and ventilatory responses to arousals (8). These findings indicate potential gender-related variations in how obesity impacts SDB in mice.

At PRISM Lab, University of Bologna, sleep apnea was studied in various mouse models of human diseases. Researchers used an algorithm that enables sleep scoring based solely on the ventilatory signal recorded in whole body plethysmography, without the need for implanted devices in the animals (9). Central sleep apneas (CSA) were distinguished from OSA (10).

Additional basic research seeks to determine if there are gender disparities in sleep-related breathing disorders in adults as a result of perinatal exposure to different types of stress (chemical, behavioral, and social). This line of research has become increasingly relevant as awareness has emerged that the impacts of perinatal stress vary by gender (11), and that these impacts are particularly significant on the hippocampus (12), a brain region which plays a central role in mediating the long-term effects of stress (13). Recent unpublished preliminary data indicates that females may be more sensitive than males to perinatal exposure to chlorpyriphos, an organophosphate pesticide, leading to a higher prevalence of sleep apneas in adulthood [Bologna PRISM Lab ongoing studies by Berteotti et al. (14)].

Another sleep disorder in which sex-related differences have recently received clinical attention is narcolepsy. Accordingly, different animal studies have examined sex-related differences in the expression of narcolepsy symptoms, particularly in terms of cataplexy severity (15–18). In 2021, a study by Coffey et al. (15) was published in Frontiers in Neuroscience, while in 2022, papers by Sun et al. (16), Piilgaard et al. (17), and Arthaud et al. (18) were published in Sleep. Across these studies, it was consistently found that females are more likely to experience cataplexy than males. According to the research conducted by Sun et al. (16), females are more likely to develop cataplexy earlier when considering the time gap between the initial degeneration of hypocretinergic hypothalamic neurons and the onset of symptoms. According to Arthaud et al. (18) cataplexy in females shows variations linked to the estrous cycle, reaching its peak during the estrous phase. These findings are salient as they consistently demonstrate gender variations in the expression of cataplexy. Additionally, animal models provide a means to investigate the potential mechanisms underlying these variations. A first step in this direction is the discovery that the hypothalamic expression of hypocretin (Hcrt) is elevated in females compared to males, as evidenced by measurements of either pre-pro Hcrt mRNA expression (19) or radioimmunoassay (20). Furthermore, the expression of Hcrt receptors differs in males and females, both in central and peripheral tissues (21). However, further studies are warranted to evaluate the role of sexual hormones implicated in the pathophysiological mechanisms of these disorders. As data obtained from human subjects do not currently match the findings from animal models, it is imperative to examine whether this discrepancy may be, at least partly, attributable to the presence of various confounding factors in human research.

As Schmidt and Bassetti highlighted in their review (22), there are differences in the methodology used in the abovementioned studies. Mouse models were not the same for all the studies, sleep scoring was performed using different time windows, and cataplexy was in some cases spontaneous, in others induced by chocolate and motor activity. This inhomogeneity likely explains why certain specific aspects, such as variations between males and females in the duration of episodes, the number of cataplexy episodes, the presence of behavioral arrests other than cataplexy (known as delta attacks), and transitions between states, vary across different publications. The discrepancies observed do not necessarily hinder the mouse model's efficacy in examining gender disparities in sleep disorders. However, they underline that methodological inconsistencies can yield varied results, emphasizing the importance of adopting standardized approaches among researchers.

## Gender differences in sleep disordered breathing: from diagnosis to therapeutic adherence

OSAS is a sleep disorder characterized by repeated episodes of upper airway obstruction during sleep, leading to disruptions in normal breathing patterns. This condition can result in disabling daytime symptoms including EDS, disturbed sleep, and long-term health consequences such as cardiovascular and metabolic diseases and cognitive impairment (23–25).

Although historically thought to primarily affect men (26–28), recent studies suggest that the average prevalence of OSAS is 27% in men and 22% in women (29, 30), resulting in a less pronounced difference in the ratio of male-to-female patients, which is ~1.5:1. Moreover, the disparity in OSAS prevalence between genders tends to narrow with age (28), as women face an increased risk during perimenopause and menopause, independently of confounding factors like age or BMI (31).

Classic symptoms of OSAS such as snoring, witnessed apneas, and sleepiness may be less prominent in women (32), who are more likely to report so-called atypical OSAS symptoms such as insomnia, fatigue, anxiety and depression, tiredness, and morning headaches (32) even in the absence of typical symptoms. This may lead to underdiagnosis of OSAS or a considerable delay in diagnosis (26), as healthcare providers may not always associate these symptoms with OSAS.

In addition, when women report these symptoms, they are often attributed to menopausal and/or mood disorders, which are potential comorbidities of OSAS (32).

A significant emphasis is placed on atypical features when attempting to cluster symptoms, polygraphic data, and clinical/comorbid features of patients with OSA. Several clustering efforts have been undertaken, often linking women to symptoms like insomnia (33). Furthermore, a cluster analysis of the extensive European OSAS patient cohort, ESADA, revealed the presence of eight specific OSAS subgroups (34), including two that are exclusively comprised of female subjects. *Cluster 8*, representing a minority of subjects, showcases a classic portrayal of the disorder, with older, obese individuals who have severe OSAS and cardiometabolic comorbidities, while *Cluster 7* (10% of the total), is distinctive for its nuanced clinical and comorbid features, comprising middle-aged women with mild OSAS and without EDS.

Such atypical clinical presentations may contribute to the lower sensitivity of OSAS screening questionnaires like STOP BANG and Berlin Questionnaire, in women. These tools are more accurate in detecting severe cases of OSAS (6), as they are more likely to exhibit typical symptoms (35).

The lower likelihood of pre-menopausal women developing OSAS, or experiencing less severe OSAS compared to men, can be partly attributed to differences in the anatomy and respiratory physiology of their upper airways, which tend to shield women from obstructive events (32). In fact, women have shorter airways, smaller lungs, a smaller diaphragm (36, 37), a more peripheral distribution of fat, and a lower airway collapsibility that act as protective factors (32).

Regarding anatomy, the impact of BMI appears to vary between genders regardless of sleeping position (38). In a recent study involving 3,319 OSA patients, researchers conducted a mediation analysis stratified by sex to explore the impact of BMI on AHI in relation to OSA development (39). In men, the impact of BMI on AHI was partially mediated by a reduction in upper airway stiffness, a decrease in circulatory delay, and an increase in arousal threshold. In women, the effect of BMI on AHI was only partially mediated by a reduction in circulatory delay.

In women, polygraphy of OSAS reflects these characteristics showing a lower AHI, shorter apneic episodes, reduced positional OSA (40), clustering of apneas during REM sleep (41), and prolonged nasal flow limitations indicating partial upper airway obstruction (42). Men and women display substantial sex differences in AHI during NREM sleep, with no significant variations during REM sleep. These differences have been linked to factors like upper airway collapsibility, arousal threshold, and ventilatory control of breathing (43), suggesting that neuromuscular compensatory mechanisms, rather than simple anatomical differences (which would manifest in all sleep states), distinguish the likelihood of OSA in men.

On the other hand, a smaller diaphragm could also contribute to a higher likelihood of experiencing sleep apnea during REM sleep (6, 37). Moreover, prolonged partial upper airways obstructions that do not meet the criteria for hypopnea nor apnea (42) are more common in women and could account for their lower apnea-hypopnea index (AHI) compared to men in NREM sleep (44). In fact, incorporating more liberal desaturation criteria or arousals narrowed the differences in AHI between sexes, suggesting stricter desaturation criteria contribute to under-estimation of OSA in women compared to men (43). Additionally, sex-related hormones such as progesterone, in conjunction with estrogens, serve a protective role by dilating airway muscles, such as the genioglossus muscle, and reducing airway resistance (45, 46). Furthermore, women may benefit from a lower metabolic rate, decreased respiratory drive instability, and diminished chemoreceptor responsiveness, which could serve as protective factors, especially for mixed or central apneas (47, 48).

Modifications in anatomy and physiology during pregnancy and menopause, two unique stages of a woman's life, can shed light on why these conditions are associated with an increased risk of OSAS, with a crucial role played by estrogens and progesterone level alterations.

During pregnancy, anatomical changes such as weight gain and fluid retention favor airway collapsibility. Physiological changes like higher progesterone levels, increased activity of upper airway dilator muscles, heightened chemo-responsiveness, and a tendency to sleep on one's side during the last trimester (32), play a protective role. Nonetheless, the occurrence of OSAS during pregnancy raises additional risks for both for the mother and the fetus, including an elevated likelihood of gestational diabetes, preeclampsia, eclampsia, fetus growth alterations (49), labor complications (44), and risks for the newborn (50).

On the other hand, the heightened risk of OSA after menopause can be attributed to the decline in progesterone and estrogen levels, which can no longer stabilize the upper respiratory airways, along with the shift in fat distribution and increased collapsibility of the upper airways (32). Previous research has indicated a reduced occurrence of OSAS in women using hormonal replacement therapy (51), prompting continued investigation into its potential benefits for OSA in females, which has shown promising results (4, 52). Nevertheless, its systematic use in women with OSA is not yet recommended (53) due to the controversial trial results.

It is also important to consider how sleep fragmentation in pregnancy or menopause could be secondary to other OSAS-related factors, like increased reflux during pregnancy or vasomotor symptoms and night sweats in menopause. Therefore, expert clinicians should be cognizant that these symptoms may mask the presence of OSAS and proceed to properly investigate them (53, 54).

Diagnosing and treating OSAS is crucial, as it is associated with an increased risk of comorbidities, such as cardiovascular diseases, hypertension, and metabolic disorders like type 2 diabetes; some studies indicate that women face an even greater risk compared to men (55). A notable finding from a large-scale study showed a higher incidence of subclinical myocardial damage in women with OSAS (56, 57), associated with a higher risk of heart failure (57). REM-OSAS (AHI>30 during REM sleep) was also linked to carotid intima-media thickness independent of NREM AHI (58), potentially due to intermittent hypoxia and deeper desaturations that occur during apneas in REM sleep. Severe untreated OSAS was identified as an independent risk factor for cardiovascular mortality in women, similar to men, in a prospective study (59) comparing treated and untreated individuals. Adequate continuous positive airway pressure (CPAP) use may help reduce this risk.

Insomnia, a frequent comorbidity of OSAS (COMISA, Co-Morbid Insomnia and Sleep Apnea), is linked to higher rates of all-cause mortality (60). With the recent surge in long-COVID cases following the pandemic, it is crucial to consider the co-occurrence of chronic fatigue syndrome when assessing sleep disorders in women (61, 62). Insomnia and fatigue should be considered when assessing symptoms and overall health impact in women with OSAS, especially when exploring therapeutic options and adherence. For instance, women with COMISA might be less compliant to CPAP and may benefit from cognitive-behavioral therapy (33, 63).

While lifestyle modifications such as monitoring weight, avoiding alcohol and sedatives, and using positional therapy are important components of OSAS management, research indicates that weight loss may not be as successful in reducing obstructive respiratory events in women compared to men (64). Currently, CPAP therapy is the most effective treatment for moderate to severe OSAS in both men and women, as it prevents the upper airways from collapsing during sleep (65). CPAP use can improve overall quality of life, EDS, and symptoms of anxiety and depression (66). Furthermore, CPAP was shown to lower diastolic blood pressure in women (67) and to reduce the risk of stroke and coronary heart disease (67, 68), although no significant findings regarding metabolic parameters have been identified thus far. The risk of using low-pressure CPAP in women must be considered (69), as the goal should be to treat OSAS by eliminating partial obstruction as well (70).

Recent studies confirm that women with OSAS (71) can adhere well to CPAP therapy, with an average compliance rate of about 80% and an average usage of 6 h per night. Research indicates that women may discontinue CPAP therapy because of factors like older age, the use of psychotropic drugs (71), and mood disorders. Although female gender is a potential risk factor for CPAP discontinuation (72), the effectiveness of CPAP therapy in women gives remains consistent (73).

It is important to note that women may require a longer duration of treatment during the night compared to men to ensure that positive airway pressure (PAP) is applied during the later part of the night when REM sleep is most prevalent (6). Mandibular repositioning devices are a viable alternative to CPAP therapy; they tend to be more effective in women than in men (74), regardless of OSAS severity, and can reduce systolic blood pressure (53).

Understanding these sex differences is essential for the development of precision and personalized medicine. It is imperative to consider gender-specific features of OSAS to properly recognize symptoms, conduct an accurate diagnostic evaluation, and provide effective treatment.

## Sex differences in clinical features of narcolepsy type 1 and type 2

Narcolepsy is classified as a rare sleep disorder, with a prevalence of 200–500 cases per million in Europe and North America (75, 76). According to some gender studies, the incidence has been found to vary, from slightly higher in males (76–79) to higher in females (80, 81). Although sex and gender differences are well-described for other sleep disorders, data for sex-specific differences in human narcolepsy are scarce (22). Sex-specific differences in human narcolepsy are even less well-studied than narcolepsy in animals (16–18), but present some noteworthy similarities.

Some studies suggest that the first symptoms of cataplexy or excessive daytime sleepiness (EDS) appear at different ages in the two sexes. For instance, Mayer et al., in a retrospective analysis (82), found that the onset of EDS and cataplexy appear to occur earlier in women than in men. Similarly, another retrospective study conducted by the European Narcolepsy Network (EU-NN) involving a sample of 1,099 narcolepsy patients with cataplexy, demonstrated that women experienced the onset of EDS at a younger age, with an average onset age of cataplexy of 25.8 years. The distribution of cataplexy onset age was found to be normal in both genders (78). Furthermore, regardless of country or time of diagnosis, women of all age groups experienced a greater delay in diagnosis (M = 15.6 years) compared to men (M = 13.8 years). Moreover, women had a significantly shorter mean sleep latency during the Multiple Sleep Latency Test (MSLT) and a significantly higher number of sleep onset REM periods (SOREMPs), which is consistent with findings from other studies (79, 83).

A later study by Won et al. (83) found that men and women reported similar degrees of subjective sleepiness as measured by the Epworth Sleepiness Scale (ESS). However, women demonstrated significantly more severe objective sleepiness on the MSLT. Both sexes presented similar narcolepsy-related symptoms, yet diagnosis was more likely to be delayed in women (78). According to the findings, 85% of men were diagnosed ~16 years after the onset of symptoms, while women tended to be diagnosed after about 28 years (83). Conversely, a retrospective study of 100 narcolepsy patients (56 males and 44 females) from the Innsbruck narcolepsy cohort (84) found no significant differences between males and females in terms of the onset age of symptoms, time-lapse between initial symptoms and diagnosis, as well as the presence and severity of cataplexy, sleep paralysis and hypnagogic hallucinations. The median age of symptom onset was 20 years, with an average diagnostic delay of 6.5 years; however, there were no differences in diagnostic delay regarding cataplexy status and gender. Moreover, this study revealed that women had higher levels of EDS compared to men (84), as measured by the ESS.

Regarding polysomnographic features of patients with narcolepsy, women exhibited greater total sleep time (83), improved sleep efficiency (78, 83), reduced Stage 1 Non-REM (NREM) sleep, extended Stage 2 NREM sleep and longer SWS duration compared to men (78). Furthermore, a recent meta-analysis on polysomnographic features of narcolepsy revealed that a higher proportion of male patients across different studies experienced fewer awakenings at night compared to healthy controls (85). That is, female narcolepsy patients were more likely to experience increased awakenings compared to their male counterparts (85). This finding is consistent with a prior population-based study by Jonasdottir et al. (86). Nevertheless, the mechanisms underlying this gender disparity in the number of awakenings among narcolepsy patients remain unknown.

The neurobiological underpinnings of sex differences in narcolepsy are complex and multifaceted. While the exact mechanisms are not fully elucidated, research suggests that both neurotransmitter function and hormonal factors contribute to the observed variations in the expression and severity of narcoleptic symptoms between males and females. For example, basic science has shown that females have a higher expression of Hcrt in the hypothalamus (19). There are also gender variations in the way Hcrt receptors are expressed in both central and peripheral tissues (21). Additionally, the expression and fluctuations of female sex hormones throughout the estrous cycle affect sleep-wake patterns (87, 88).

In conclusion, substantial evidence indicates sex-specific variations in the manifestation of narcolepsy symptoms. This observation is particularly evident in animal studies, where a more severe form of cataplexy and an earlier onset age in females is observed. In contrast to the animal data, clinical studies have not yet confirmed the significant impact of sex on human cataplexy severity, underscoring the need for more systematic and controlled clinical studies. In a recent study utilizing the EU-NN database, an unsupervised machine learning approach involving agglomerative hierarchical clustering was applied to identify four distinct clusters of narcolepsy patients with cataplexy (89). For instance, Cluster 4 exhibited a strong female predominance, along with a combination of mild cataplexy, hypnagogic hallucinations, and sleep paralysis, which may suggest a female-specific narcolepsy subtype. However, a cluster with more severe cataplexy, primarily affecting females, was not identified.

Nonetheless, the limited clinical data indicate that the initial symptoms of EDS tend to manifest at a younger age in women, and that it is more severe in women than in men. Additionally, there is a significant delay in diagnosing narcolepsy in females compared to males, with a prolonged interval between symptom-onset and diagnosis. Further research is needed to explores sex disparities in human narcolepsy in order to improve our understanding of how sex impacts narcolepsy symptoms and sleep patterns. Notwithstanding, these studies shed light on the nuanced features of narcolepsy, emphasizing the importance of considering both sex and age when addressing symptoms and tailoring interventions for narcolepsy patients.

## Gender differences in restless legs syndrome

RLS is a common neurological disorder characterized by an irresistible urge to move the legs usually accompanied or caused by unpleasant sensations. Symptoms begin or worsen during periods of rest or inactivity, especially in the evening and at night, and can only be alleviated by moving the legs (90). RLS is a distressing condition that interferes with rest and impairs sleep quality, affecting the physical and mental health of both men and women (91, 92). Conversely, periodic leg movements during sleep (PLMS) consist in nighttime myoclonus that disrupts sleep due to involuntary repetitive leg and/or arm movements (93).

Females experience RLS at a rate twice as high as males across all age groups and populations, with an average prevalence of 4.6% in men and 8.6% in women (94). Possible explanations for this gender discrepancy include iron levels and sex hormones, particularly during specific periods of a woman's life, such as menopause or pregnancy (94). Women seem to experience their RLS symptoms as more distressing than men, as shown by validated scales [International Restless Legs Syndrome study group severity rating scale (IRLS); Restless Legs Syndrome-6 scale (RLS-6); Clinical Global Impression of Severity (CGI-S)], while PLMS indices tend to be significantly higher in men (95). These findings suggest a possible gender difference in the phenotypical presentation of RLS, manifesting with predominantly sensory symptoms in women and motor symptoms in men. Also, women and men perceive the burden of RLS symptoms differently in (95). In 536 women with RLS, the perception of symptom severity did not consistently change with variations in female hormonal profiles. Among non-menopausal women, 29% perceived increased RLS severity during menses, while 23% of the total cohort declared worsening of symptoms during pregnancy, and 69% reported an exacerbation of symptoms after menopause (96). Pre-menopausal patients had significantly higher mean RLS severity scores compared to menopausal women, reflecting a higher risk of iron deficiency among pre-menopausal women. It is therefore conceivable that the increased likelihood of developing comorbidities with age may interfere with the accurate perception of RLS symptom severity in elderly patients (96).

Seasonal changes in RLS symptoms might be observed, with symptoms worsening, for instance, during the summer months only in men (97). Men typically exhibit increased sweating and overall sudomotor activity compared to women, primarily as a result of elevated body temperature. Thus, it might be theorized that dysregulated thermoregulation, coupled with the altered autonomic nervous system responses in RLS patients, may account for the exacerbation of RLS symptoms in men during the summer (97). At the same time, hormonal status, insomnia, and iron deficiency could play a significant role in the pathogenesis of RLS in women, with these factors being less affected by seasonal changes (97).

A significant correlation was observed between RLS and hypertension in a large cohort of 673 treated hypertensive subjects. The study showed that individuals with frequent RLS (≥2 episodes/week) combined a PLMS index ≥26/h are at a higher risk of developing resistant hypertension (98).

RLS may also affect the 24-h blood pressure (BP) profile. To our knowledge, currently only one study in the literature has shown that patients with RLS have higher nighttime systolic BP than controls (99). Further clinical studies are necessary to validate these findings, with a specific emphasis on monitoring BP during objectively defined sleep, the impact of leg movements during sleep, alterations in sleep architecture, and the potential cardiovascular risks for both genders. In fact, the cardiovascular risk could manifest differently between genders, as documented in a cross-sectional observational study enrolling 4,080 participants from the Sleep Heart Health study. RLS was present in 6.8% of women and 3.2% of men; in gender subgroup analyses, the association between RLS and hypertension persisted only in women (100).

Several studies also suggest RLS may be linked to key components of the metabolic syndrome, including diabetes, obesity, and dyslipidemia. Vitamin D levels seem to correlate with the frequency and severity of RLS (101). Women with RLS are more likely than controls to present dysthyroidism, usually developing hypothyroidism after the onset of RLS. RLS in pregnant women is associated with a higher prevalence of thyroid disease, and both subclinical hypothyroidism and RLS independently contribute to adverse fetal/maternal outcomes (102).

With respect to neuropsychiatric status, individuals with RLS exhibit a higher incidence of depressive symptoms, depression, and suicidal thoughts/risk compared to controls, particularly among females, younger individuals, and those with insomnia (103). Attention-Deficit/Hyperactivity Disorder (ADHD) symptoms are typically more frequent in boys during childhood, equally distributed in young adults, and more prevalent in elderly women. The relationship between RLS severity and ADHD is significant primarily in females, suggesting that the impulsivity and hyperactivity subcomponents of ADHD, which are more pronounced in males, decrease to undetectable levels with age. Conversely, the inattentive symptoms of ADHD are more commonly observed in females and tend to worsen with age, becoming more prominent (104).

## Discussion

The brain, a sexually dimorphic organ, undergoes distinct processes of maturation and aging in men and women. Sex hormones play a key role in this process, as evidenced by the varying sleep patterns during different reproductive phases in females. Animal models offer enhanced accuracy and enable clearer differentiation between various estrous stages. For example, they demonstrate a connection between female cataplexy and the estrous cycle, as well as a susceptibility of select brain regions, namely the hippocampus, to perinatal stress, resulting in apneas later in life. This potential over-reactivity of the female brain may be linked to the later development of Alzheimer's disease in women. In fact, studies have consistently shown that women have a higher risk of developing this neurodegenerative condition, and hypoxia is a major contributing factor (105).

Important behavioral differences impact the occurrence of EDS in women and men in different disease models. In narcolepsy, women seem to experience more EDS than men, with symptoms appearing earlier. However, in OSAS, women have more disrupted sleep but less EDS than men, leading to a higher incidence of COMISA. This may be partly attributed to a higher Hcrt expression in the female hypothalamus (19), as well as a dimorphic expression of both central and peripheral Hcrt receptors (21). Additionally, there is a sex-dependent response to hypoxia, particularly in REM sleep.

In terms of narcolepsy and OSAS, women are consistently met with delays in accessing care and obtaining a diagnosis compared to men. This postponement may be related to their social roles and work ethic. They indeed have significant household responsibilities within the family, thus feeling obligated to manage both sleepiness and interrupted sleep. Nevertheless, women suffer significant health consequences and stigma throughout their lives. This is especially true for OSAS, which poses potential risks for both the mother and fetus during pregnancy, as well as significant cardiovascular risk in the post-menopausal phase compared to men (57, 59).

RLS, instead, is specifically linked to insomnia, usually with minimal sleepiness symptoms, and is significantly more prevalent in women, at least after puberty (94). The severity and health consequences of RLS are also more disabling for women than men, with hormone-related exacerbations throughout their reproductive years. Interestingly, the phenotypic, clinical and polygraphic aspects of RLS vary between genders, with motor (95) and autonomic (97) symptoms prevailing in men. Among RLS female patients, depression (103) is a frequent comorbidity, as seen in most types of insomnia (106); attention deficits (107) are also common, as are behavioral changes in elderly women, where RLS may play a role in sundowning (108).

Depressive symptoms and increased fragmented sleep have significantly impacted the treatment efficacy and adherence of women with various sleep disorders, spanning from CPAP (72) to RLS. Iron and vitamin D deficiency are major but often overlooked factors in women (101).

Finally, iatrogenic effects from antidepressants are more frequent and severe in women compared to men. Women are also more susceptible to the adverse drug reactions of hypnotics, including the potential occurrence of Sleep-Related Eating Disorder (109) and fractures from nocturnal falls (110).

Therapeutic approaches that address the brain's dimorphic nature, such as cognitive-behavioral therapy-adjuvant treatments, dual orexin receptor agonists, and iron and vitamin supplementation, have the potential to improve quality of life and prevent cognitive impairment in women. Increased focus and allocation of healthcare resources are necessary to ensure prompt and sustainable diagnosis of sleep disorders in females, with special consideration for atypical phenotypic variations. Moreover, it is imperative to promote and maintain early and ongoing treatment, particularly during pregnancy and the menopause transition, as these are times when sleep disorders have a significant impact on women's mental and physical wellbeing.

## Conclusions

Drawing from the existing evidence, this review emphasizes the importance of conducting clinical and basic science studies to enhance understanding and awareness of gender differences, especially in the field of sleep medicine.

Understanding the differences in sleep disorders between men and women in terms of prevention, clinical signs, treatment approaches, prognosis, and psychological and social impacts is crucial for shaping future therapeutic strategies. Risk factors for sleep disorders impact men and women differently, while clinical manifestations are often responsible for delayed diagnosis in women. The development of new drug therapies should take into consideration potential gender-related variations in pharmacokinetics and pharmacodynamics, as these could influence the efficacy of treatments (111, 112).

To advance our understanding of gender differences, the field of sleep medicine must prioritize conducting trials that explore the unique pathophysiology of diseases, the distinct clinical manifestations observed in women, and the varying treatment outcomes between males and females.

## Author contributions

EP: Conceptualization, Funding acquisition, Methodology, Project administration, Supervision, Validation, Visualization, Writing – original draft, Writing – review & editing. RS: Writing – original draft, Writing – review & editing. EB: Writing – original draft, Writing – review & editing. MD: Writing – original draft. MF: Writing – original draft, Writing – review & editing. FP: Writing – original draft, Writing – review & editing. GZ: Writing – original draft, Writing – review & editing. CL: Funding acquisition, Project administration, Validation, Writing – original draft, Writing – review & editing.
